# Scotch Physicians and Surgeons

**Published:** 1888-05-05

**Authors:** C. G. Furley


### Scotch Physicians and Surgeons.

By C. G. Furley.

(*Continued from p.* 41.)

### JAMES SYME.

It was unfortunate that the two young men had the same ambition, and were pursuing their ends by the same paths. Liston, while first demonstrator and then lecturer on anatomy, had been cultivating surgery, and securing renown as an operator of assured skill, invention, and resource. Syme did the same; therefore it was inevitable that the day should come when he could no longer act as Liston's subordinate, but must shine forth as a rival. Only in the case of exceptionally noble natures can such things happen without some bitterness of feeling; and, without any quarrel, professional rivalry brought about an estrangement between Syme and Liston which lasted till the removal of the latter to London gave to each "ample room and verge enough" to pursue their work without encroaching on each other's prerogative.

This coolness, however, did not come till a later day. In 1823, when Syme became a Fellow of the Royal College of Surgeons of Edinburgh, he dedicated his inaugural thesis, on Necrosis, "to his friend, Robert Liston, Esq."; and when, in October of the same year, Syme performed the operation of amputation at the hip-joint, which had never before been attempted in Scotland, Liston was his chief assistant.

In the *Edinburgh Surgical and Medical Journal* for January, 1824, he gives a full account of this operation, which it is not necessary to enter into here, though I will allude to one point. He intended to carry out the amputation after the method of Lisfranc, which he had seen in Paris, but finding after he had made the first incision that the cramped position which, from long habit, the patient's leg retained, made this impossible, he changed his plan on the instant, and without removing the knife, made a variation on Lisfranc's operation, which was perfectly successful. As the whole amputation did not take a minute, there could not have been a moment's hesitation in resolving on the change of plan. It is such readiness that marks genius in surgery.

It may also be well to speak a word as to the remarkable speed with which the operation was accomplished. The public, reading an account of some surgical operation, pictures a scene of prolonged horror, and does not realise that these incisions and so forth are made with as much assured speed as an experienced artist drawing a line or a skilful sempstress making a stitch. Some operators are indeed slower than others; but the slowest are swift, and the swiftest attain a speed that is bewildering. On this point I may tell an anecdote about an Edinburgh surgeon who is still living. A French doctor—so the story goes—who had heard of the speed and brilliancy of his operations, came to see one performed, and to take notes of the method. Armed with book and pencil he took up a convenient position near the operating table; but just as the Scotch surgeon was about to commence his work the French spectator dropped his pencil. He stooped to pick it up, and having done so, turned his eyes again on the table in time to see the surgeon lay aside his knife. The operation was over; in that one instant all had been done.

The story may not be free from a touch of the apocryphal; but at the most it is but an exaggeration of a skill and swiftness which to the uninitiated seem miraculous.

It was in 1824 that Syme's connexion with Liston's anatomical school ceased. It placed him in a difficult position, as it took away from him the foothold he had already won on the professional ladder, without showing him where to find a substitute. He was not rich in friends who had the power to help him, nor had he money to enable him to pause and look around for an eligible way of earning his living, his father, who had died two years before, having left little more money than would pay his debts. Still, it would have been impossible to Syme to give up his ambition and decline to a lower level of professional effort. He had cast in his lot with the science of surgery, determined to be a teacher and an operator, and to do well in both capacities; and he would do no other work.

In these circumstances he ventured to follow Liston's bold footsteps so far as, in turn, to set up an extra-mural school. He was joined by three friends, Drs. Mackintosh, Argyle Robertson, and Fletcher; and they rented a house in Brown Square, very nearly at the back of the University, where Syme taught anatomy, practical anatomy, and surgery. The venture was fairly successful; he had fifty students the first session he taught, and his reputation increased. But a circumstance, trifling in itself, caused him to break again from this association, as from previous ones. His old schoolfellow, classmate, and friend, Dr. (afterwards Sir Robert) Christison, having become a candidate for the chair of medical jurisprudence, Syme gave a testimonial as to his fitness to fill it. His doing so offended his colleagues at Brown Square, who thought he should have consulted them before doing so, a piece of interference with his private opinions and actions which Syme resented to the point of throwing up all connexion with them and the Brown Square school. And so, once more, he cast away the advantages of a recognised status.

The step was a rash one, but it answered well, for it enabled him to give up the teaching of anatomy and devote himself wholly to surgery.

He was sufficiently well known to have the right to expect a fair amount of success; but he felt that one thing was lacking to him. He required the advantage of a hospital appointment—the status it gives in the eyes of the laity, and, more than that, the opportunity of demonstrating his operations to his students in a proper theatre. Hitherto they had all been done in private, where they could be seen by comparatively few, and could not have the éclat of hospital work. He therefore asked the managers of the Royal Infirmary to appoint him one of the surgeons there. Under ordinary circumstances the appointment would have been given as soon as it was asked for, but Liston was one of the infirmary surgeons, and the alienation between him and Syme was matter of public knowledge. The managers fearing, therefore, that their being brought into contact, as they inevitably would be if both belonged to the same institution, might end in a quarrel which would discredit them both in the eyes alike of the public and their students, declined to appoint him in the meantime.

Defeated again, Syme was not baffled. If he could not secure an entry to the infirmary he would have a hospital of his own. So, with a view to converting it into a surgical hospital, he rented Minto House.

Minto House exists no more; it has been pulled down in the course of some improvements of the city; but its site is occupied by a building which still bears the name of the Minto House School of Medicine. It stood, however, till a few years ago, in what is now called Chambers Street, a broad thoroughfare that runs from the South Bridge to George IV. Bridge, along the side of the University and the front of the Industrial Museum. It was a large square house, once the town residence of a famous Border family, the Elliots of Minto, one of whose daughters gave the world that immortal strain of sorrow, the ballad called "The Flowers of the Forest." A century and a-half ago, Minto House stood in the most aristocratic part of Edinburgh, on the brow of the hill overlooking the Cowgate, and near the Horse Wynd. The tide of gentility having, however, flowed northward and westward by 1829, Syme was able to secure Minto House, with its gloomy, untended slope of garden ground, at a comparatively small rental.

Nevertheless, for any man to start a hospital at his own risk, and his own at least initial expense, is a serious venture. It is exceptionally serious to a young man who has no money saved, and must trust to the future to repay what he expends. At this time and during the few years that followed, Syme was often in sore straits for money, and occasionally had to borrow it; but these loans were always repaid with the utmost possible promptitude.

In furnishing Minto House as a hospital for twenty patients and the necessary attendants, he calculated that the annual expense of maintenance would be about £700, besides the cost of fitting it for its new purpose, which he estimated at £300. Towards this he expected that the public and students' fees would contribute about £550, leaving the remainder, £150, to be borne by himself. As a matter of fact, his personal expenditure on the hospital during the first year of its establishment was £179, though both the public contributions and the students' fees amounted to more than had been anticipated. During the first year the expenses were naturally exceptionally heavy, and after this time the hospital could easily have been made self-supporting, and even profitable, if Syme would have admitted a larger number of students than the forty he made his ultimatum. But he felt that success in clinical teaching can be attained only by keeping the proportion between students and patients very close; otherwise, the students cannot glean that experience in the dressing of wounds which is essential to their training.

The Minto House Hospital was therefore conducted at a loss, and though the contributions of the public increased year by year, a large proportion of the expense was borne by Syme himself. In fact, in the four years during which he was connected with the hospital, more than half its cost—£1,775 out of £2,984—was defrayed by him. A man who will dare to lose so much for the sake of his art deserves to win in the end; and it may be assumed that the money was not ill-invested in bringing Syme's name and fame before the notice of the public—an Edinburgh public, keen to notice the rising men in medical science; and a wider public, that has to take such knowledge at second hand and admires very much where it is told to do so. But this was a straitened and anxious time with him, and the memory of its cares and troubles was doubtless the inspiration from which he evolved his memorable address to the medical graduates of 1852. In this he warned them to beware of incurring debts in the early years of their practice, and in connexion with this subject to avoid early and imprudent marriage. He warned them how poverty, especially when others, dearer than themselves, were involved in it, might tend to lower their ideal of honour by making them think all things lawful by which they could earn money, and degrading them to the level of quacks. On this subject he, who had felt the dear burden of a wife and family—heavier because so much beloved—during years of struggling and ill-rewarded toil, could speak with all right and earnestness as one who, having been tempted, had *not* fallen.

*(To be continued.)*

